# Disparities in care and outcomes for adolescent and young adult lymphoma patients

**DOI:** 10.1002/jha2.797

**Published:** 2023-09-27

**Authors:** Allison Rosenthal, Adam Duvall, Justine Kahn, Niloufer Khan

**Affiliations:** ^1^ Mayo Clinic Arizona Division of Hematology Medical Oncology Phoenix Arizona USA; ^2^ Department of Medicine Section of Hematology/Oncology University of Chicago Chicago Illinois USA; ^3^ Department of Pediatrics Division of Pediatric Hematology/Oncology/Stem Cell Transplantation Columbia University Medical Center New York New York USA; ^4^ Department of Hematology and Hematopoietic Cell Transplantation Duarte City of Hope Duarte Canada

**Keywords:** AYA lymphoma populations, DLBCL, HL

## Abstract

Though survival outcomes among adolescents and young adults (AYAs) with lymphoma have improved over the last three decades, socially vulnerable populations including non‐White, low‐income, and publicly insured groups continue to trail behind on survival curves. These disparities, while likely the result of both biological and non‐biological factors, can be largely attributed to inequities in care over the full cancer continuum. Nationally representative studies have demonstrated that from diagnosis through therapy and into long‐term survivorship, socially vulnerable AYAs with lymphoma face barriers to care that impact their short and long‐term survival. Thus, improving outcomes for all AYAs with lymphoma requires dedicated study to understand, and then address the unique challenges faced by non‐White and low‐income lymphoma populations within this age group.

## 1 INTRODUCTION

**FIGURE 1:**
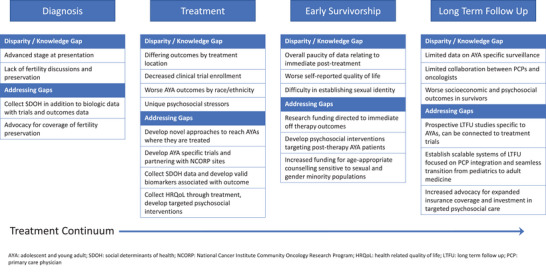
Disparities in adolescent and young adult (AYA) lymphoma.

Despite continued improvements in adolescent/young adult (AYA) lymphoma survival, outcome disparities persist. Even after consideration of factors thought to drive disparities (e.g., distance‐to‐care, treatment location) [1], non‐White (vs. White) patients are twice as likely to present with advanced stage and are over 3‐times more likely to die post‐relapse [2]. Recent efforts to explain these disparities have focused on factors influencing care over the cancer continuum. These include under‐insurance, the likelihood of timely diagnosis, differences in treatment location, clinical trial enrollment, receipt of guideline‐concordant survivorship care, and the unique psychosocial needs of the AYA population (Figure 1).

The most frequently diagnosed lymphomas in AYAs are Hodgkin lymphoma (HL) and diffuse large B‐cell lymphoma (DLBCL) [3]. Available data on disparities in AYA lymphoma patients predominantly revolve around these two subtypes and persist throughout the entire lymphoma journey, including diagnosis, treatment, transitions of care, early survivorship, and long‐term follow‐up.

Inequities observed in therapy are also observed in survivorship and efforts to ensure consistent long‐term follow‐up, particularly in minority and low‐income populations, are critical. System‐level factors, including lack of health insurance, transportation barriers, and information needs have been cited as barriers to care in adolescent survivors [4, 5].

### 1.1 Diagnosis and treatment

AYA individuals diagnosed with lymphoma often face delays in diagnosis due to a combination of factors. These include a lack of consideration for cancer in this age group, limited access to health care due to social determinants of health (SDOH), and insurance‐related obstacles. Unfortunately, these delays can result in more advanced‐stage presentations, significantly impacting patient outcomes.

Diagnostic delays are a known challenge for AYAs with lymphoma. In one study of AYAs treated for HL in New York State, Kahn et al. found that non‐White patients were more likely to present with advanced‐stage disease, which is often considered an indicator of access to primary health care. In a registry analysis of 58,000 AYAs from Keegan et al, having public or no insurance was associated with progressively higher odds of late‐stage presentation for nearly all cancer sites. Among patients with HL, this resulted in a two‐times increased risk of death overall [6]. In another study of patients with Burkitt lymphoma, Alvarez et al. identified an association between low socioeconomic status and elevated risk of death among young adult patients, suggesting that delays in diagnosis for this highly aggressive but very curable tumor likely contributed [7].

Adolescent and young adult patients with lymphoma confront a range of other unique challenges that significantly impact their healthcare journey. Despite the provisions of the Affordable Care Act, which expanded Medicaid and allowed young adults to stay on their parents’ insurance plans until age 26, AYAs are recognized as the age group most likely to be uninsured or underinsured in the United States [8, 9]. This lack of adequate insurance coverage can profoundly influence where these patients receive their lymphoma treatment, whether care is received without interruption, and if surveillance and survivorship care guidelines are followed. The risk of infertility is another crucial consideration when treating AYA patients with lymphoma, yet discussions about fertility preservation occur inconsistently. Having these conversations upfront is associated with better patient‐doctor relationships and improved health‐related quality of life [10].

Discrete components of care after diagnosis, including treatment location, supportive care, and clinical trial enrollment are associated with optimal outcomes in AYA lymphoma. Numerous studies have found that for AYAs with lymphoma, receiving therapy at large academic cancer centers is associated with better outcomes. Using data from the California Cancer Registry, Kahn et al. reported a significant difference in the likelihood of being treated at an NCI center in HL patients with public versus private insurance [11]. Among a cohort of Medicaid‐insured patients with HL, Kahn et al. reported that AYAs were significantly less likely than children to receive treatment at National Cancer Institute (NCI) Cancer Centers, a factor that directly impacted survival risk in this group [1]. Findings suggested that efforts focused on treatment location and on improving access to an NCI Cancer Center/Children's Oncology Group (COG) center may be a key part of addressing previously observed age‐related survival differences in HL. Further, while more work is needed to understand how location‐of‐care impacts survival, potential reasons for superior outcomes at NCI facilities include higher clinical trial enrollment rates and access to tertiary‐level care during and after therapy.

While clinical trial enrollment is associated with superior outcomes among AYAs with lymphoma, disparities persist even within trials. A retrospective analysis of 1,605 patients enrolled on AHOD0031, AHOD0431, and AHOD0831 revealed that despite equal relapse rates during up‐front therapy, non‐White patients had a nearly 1.9‐fold increased risk of death overall (95% confidence interval [CI] 1.1–3.3). This survival difference was largely driven by differential hazards of post‐relapse mortality between groups. Specifically, five‐year post‐relapse survival probabilities in non‐Hispanic White patients was 90% versus 80% in Hispanic patients and 66% in non‐Hispanic Black patients. Compared with White patients, Hispanic and non‐Hispanic Black children had 2.7 times (95% CI, 1.2–6.2) and 3.5 times (95% CI, 1.5–8.2) higher hazards of post‐relapse mortality, respectively [2]. The striking difference in the risk of all‐cause mortality between White, Black, and Hispanic patients raises the possibility that differences in salvage therapy or supportive care outside of consortium clinical trials may be driving the disparities observed at the population level.

Socioeconomic status, rurality, and race/ethnicity also impact 5‐year survival outcomes for AYA cancer patients. Studies have shown that non‐Hispanic Black patients have worse long‐term survival in conditions like DLBCL and lower socioeconomic status, non‐Hispanic Black race/ethnicity, and residing in rural areas are associated with worse outcomes [12]. While disparities persist in survivorship care engagement, efforts are being made to close these gaps, particularly for Hispanic cancer survivors [13]. Additionally, disparities are observed in consistent and comprehensive follow‐up care, with non‐Hispanic Black patients and males being less likely to receive such care [14, 15].

In the case of HL, AYA patients without insurance or those with public health insurance are more likely to present with advanced‐stage disease, and the absence of insurance is linked to worse overall survival [6, 16–18]. Older young adult cancer patients are more frequently cared for in community settings, which can also introduce disparities in their health care [19, 20]. These older young adult patients are particularly vulnerable to gaps in care due to the risk of being uninsured.

These findings underscore the need for targeted interventions and healthcare policies to address disparities and ensure equitable access to quality care for AYA lymphoma patients across diverse demographics.

Historically participation in clinical trials has been low for AYA patients compared to other age groups however access to innovative treatments and improvement in outcomes can be achieved by encouraging enrollment and expanding access. Barriers to enrollment in clinical trials include limited access to trial centers, preconceived notions about clinical research, and competing psychosocial priorities [21]. In contrast, having AYA champions, frequent communication between pediatric and medical oncology and supportive research infrastructure have been shown to facilitate AYA trial enrollment [22]. To enhance outcomes for AYA lymphoma patients, it is imperative to tailor clinical trial opportunities to this age group. Increasing AYA patient participation in research studies can provide valuable insight into age‐specific outcomes, including differences between AYAs and both pediatric and older adult populations. Additionally, it can help us better understand treatment‐related toxicities, including patient‐reported outcomes that uniquely impact AYA patients, and the underlying disease biology. Recent efforts aimed at inclusive trial design involving pediatric and adult cooperative groups show promise in addressing these challenges.

Variations in biology may also underlie discrepancies in treatment‐related outcomes among Hodgkin lymphoma patients receiving pediatric versus adult protocols, especially in the emerging young adult population (age 12–21). A comparative analysis focused on Hodgkin lymphoma patients aged 17–21, treated with protocols E2496 and AHOD0031, demonstrated superior 5‐year failure‐free survival (FFS) and overall survival (OS) rates for patients treated on the pediatric protocol. Specifically on protocol E2496, the 5 years FFS was 68% with an OS of 89% in contrast to 81% FFS and 87% OS for patients on the COG protocol (*p* = 0.001) [23].

The financial challenges of cancer treatment can be especially daunting for young adult patients, who are less likely to have stable careers or comprehensive insurance coverage compared to older adults. Expenses related to living with parents or being parents themselves, determination to achieve educational career or relationship milestones, and financial hardships related to student loans and the lack of accrued sick leave can all contribute. AYAs are particularly vulnerable to financial toxicity due to high out‐of‐pocket costs, inadequate health insurance, job constraints, disruptions to education or work, limited assets, and limited experience navigating the healthcare system. Notably, fertility‐related financial concerns can compound this burden. At Memorial Sloan Kettering, a pilot study introduced universal financial toxicity screening using the Comprehensive Score for Financial Toxicity. This approach helped identify at‐risk patients and direct them toward appropriate support aiming to alleviate the financial burdens of cancer treatment [24].

While receiving treatment, AYAs also grapple with unique psychosocial concerns including body image, relationships, and disruptions to their work, education, and career plans. Without dedicated efforts and age‐appropriate interventions, many AYA patients may lack the coping strategies needed to navigate these challenges. Finally, AYA patients often find themselves in healthcare settings designed for older adults or children, contributing to feelings of misunderstanding and isolation [25].

Psychosocial stressors and division of care between pediatric and adult centers result in variable access to age‐appropriate healthcare for AYAs with cancer [26]. A recent dataset comparing healthcare utilization in AYA lymphoma patients to older patients with lymphoma showed that AYA patients with aggressive lymphoma (mostly DLBCL and HL) are almost twice as likely to be at higher risk of significant acute care usage. Acute care was frequently sought for concerns related to infection and pain but notably 8.8% presented with substance use or psychiatric‐related concerns, highlighting the importance of early inclusion of palliative medicine and psychosocial support [27].

### 1.2 Transition to early survivorship

Inequities observed in therapy are also observed in survivorship and long‐term follow‐up and efforts to ensure consistent long‐term follow‐up, particularly in minority and low‐income populations, are critical [4, 5, 28] System‐level factors, including lack of health insurance, transportation barriers, and information needs have been cited as barriers to care in adolescent survivors [5]. However, there is a relative paucity of data on disparities among AYA patients in transition to early survivorship and long‐term follow‐up, and more research is needed.

As patients end active treatment and enter the survivorship phase of care, the frequency of visits with their primary oncologist decreases, and patients may feel disconnected from their providers. In national surveys, only 32% of oncologists discussed who patients should see for follow‐up care, and < 5% provided written survivorship plans to their patients [29]. Seventy percent of AYA survivors continued to identify their oncologist as the most important provider for their health care, and 41% reported low confidence in managing their survivorship care. On multivariate analysis, lack of confidence was associated with non‐White ethnicity and lack of a survivorship care plan (*p* < 0.05) [30]. Provision of treatment summaries and survivorship care plans to early survivors can improve their confidence and understanding of their health care and help them to advocate for themselves in the future.

Following cancer treatment, AYA survivors report worse physical and emotional health‐related quality of life, when compared to peers without a history of cancer [31]. Like their peers, younger AYA survivors note difficulty with establishing their identity, developing a positive body image and sexual orientation, and separating themselves from their parents. Fifty‐seven percent of AYAs reported an unmet need for information, 41% reported an unmet need for counseling and 39% felt unsatisfied with their level of practical support [32]. They may feel left behind when compared to their healthy peers. Social support, maintaining social relationships, and a sense of normalcy are an issue of major concern for AYA survivors [33]. Support for maintaining sexual health and fertility has also been identified as an area of unmet supportive care by AYA survivors [34].

The effects of financial toxicity during treatment extend into survivorship, as patients may struggle to pay off bills related to cancer treatment and may not experience the same levels of cognition and energy as they had prior to treatment. In the US AYA HOPE study, most AYAs (72%) in full‐time work or school reported returning to those activities [35]. In an Australian study of AYA survivors, more than half reported mild, moderate, or marked impairment with role functioning when returning to work or school [36]. Among the US population, 15% of survivors reported bankruptcy concerns/filing, 42% reported borrowing money to pay bills, and 58% reported using savings for medical bills [37]. In addition, 35% reported skipping or delaying survivorship care and 23% reported skipping or delaying treatment. These financial toxicities can be compounded by student loan debt or credit card debt, both of which are common. Fertility‐related financial concerns can add an additional financial burden for AYA survivors, with patients who experience greater financial toxicity reporting higher levels of reproductive concerns and more decisional conflict about building a family [38]. Treatment‐related financial toxicity has significant late effects and should be addressed early during therapy.

### 1.3 Long‐term follow‐up

Evidence‐based guidelines recommend that AYA cancer survivors continue receiving specialized survivorship care, for early detection of late effects and improved quality of life. However, there is little data on the specific surveillance needed by AYA survivors, with much data extrapolated from childhood cancer survivors or older adult patients. AYA patients are at substantial risk of being lost to follow‐up, as they may be transitioning between pediatric and adult care systems in addition to moving away from their treating institutions for work or school [39]. Unfortunately, only 18% of AYA survivors report receiving optimal adult‐focused risk‐based care. On a systems level, there can be limited communication between oncologists and primary care physicians, further hampering the transition [40]. Structured transition programs with communication between survivorship clinics and primary care providers are under study for this population [40, 41].

Throughout their lifetime, AYA cancer survivors are more prone to mental distress than their peers without a history of cancer. In the US National Health Interview Survey, AYA survivors were more likely to report mental distress as well as an inability to afford mental health care than a comparison group of healthy adults. Survivors with public or no health insurance reported greater distress than survivors with private insurance [42]. AYA cancer survivors are more likely to experience serious psychological distress than older cancer survivors [43]. Younger age at treatment and greater financial stress or lower income have been associated with greater distress [44, 45, 46]. Fear of recurrence is a significant challenge for AYA survivors, ranging from 31% to 85% in reported series [47]. Having Medicaid or Medicare coverage has been associated with a greater fear of recurrence [48].

Finally, and quite significantly, AYA survivors remain at risk for worse socioeconomic outcomes, even in late survivorship. In a selection of participants from the US 2008–11 Medical Expenditure Panel Survey, adults who had been diagnosed with cancer as an AYA were more likely to be insured by Medicaid, in fair or poor health, and to have a lower income when compared to adults without a history of cancer. They were more likely to report limitations in their activities and more likely to report an inability to work due to illness or disability [49]. Lower education levels correlated with reduced work ability in a survey of Norway AYA survivors [50]. AYA cancer survivors with lower education and lower annual income have a greater risk of nonadherence to survivorship recommendations [51]. These disparities persist within populations, even among groups already facing disparities. In the 2009–2018 US National Health Interview Survey, non‐Hispanic Black AYA survivors were less likely than non‐Hispanic Black age‐and‐sex‐matched controls to have a family income > 45K/year, have completed a bachelor's degree or higher, or to have private insurance [52].

## 2 CONCLUSIONS

In conclusion, despite the progress made in understanding and addressing the specific needs of AYA lymphoma patients, inequitable access to healthcare, unique psychosocial concerns, and financial toxicity continue to be significant roadblocks to achieving further improvements in outcomes for this unique patient population. These disparities encompass various aspects, including delays in diagnoses, limited access to age‐appropriate specialized care and clinical trials, challenges related to fertility preservation, psychosocial issues such as the need for peer support, insurance hurdles, communication difficulties, and survivorship care. These challenges exert a significant emotional, physical, and psychosocial toll on AYA cancer patients. Therefore, it is crucial to raise awareness about the unique needs of AYA lymphoma patients to advocate for meaningful change and improvement in their care.

## CONFLICT OF INTEREST STATEMENT

The authors declare no conflict of interest.

## FUNDING INFORMATION

Not applicable

## ETHICS STATEMENT

The authors have confirmed ethical approval statement is not needed for this submission.

## PATIENT CONSENT STATEMENT

The authors have confirmed patient consent statement is not needed for this submission.

## CLINICAL TRIAL REGISTRATION

The authors have confirmed clinical trial registration is not needed for this submission.

## Data Availability

Not applicable
